# Chitin amendments eliminate the negative impacts of continuous cropping obstacles on soil properties and microbial assemblage

**DOI:** 10.3389/fpls.2022.1067618

**Published:** 2022-11-24

**Authors:** Yanli Fan, Junjie Liu, Zhuxiu Liu, Xiaojing Hu, Zhenhua Yu, Yansheng Li, Xueli Chen, Lujun Li, Jian Jin, Guanghua Wang

**Affiliations:** ^1^ Key Laboratory of Mollisols Agroecology, Northeast Institute of Geography and Agroecology, Chinese Academy of Sciences, Harbin, China; ^2^ University of Chinese Academy of Sciences, Beijing, China; ^3^ Heilongjiang Academy of Black Soil Conservation and Utilization, Heilongjiang Academy of Agricultural Sciences, Harbin, China

**Keywords:** continuous cropping, pure and crude chitin, soil pH, potential plant pathogens, specific disease suppression

## Abstract

Continuous cropping of soybean leads to soil environment deterioration and soil-borne disease exacerbation, which in turn limits the sustainability of agricultural production. Chitin amendments are considered promising methods for alleviating soybean continuous cropping obstacles; however, the underlying mechanisms of soil sickness reduction remain unclear. In this study, soil amendments with pure and crude chitin at different addition dosages were employed to treat diseased soil induced by continuous cropping of soybean for five years. Chitin amendments, especially crude chitin, remarkably increased soil pH, available phosphorus (AP), potassium (AK) and nitrate nitrogen ( ${\text{NO}}_{3}^{-}$ -N) contents, and improved soybean plant growth and soil microbial activities (FDA). Additionally, chitin application significantly enriched the relative abundances of the potential biocontrol bacteria *Sphingomonas*, *Streptomyces*, and *Bacillus* and the fungi *Mortierella*, *Purpureocillium*, and *Metarhizium* while depleted those of the potential plant pathogens *Fusarium*, *Cylindrocarpon* and *Paraphoma*. Moreover, chitin amendments induced looser pathogenic subnetwork structures and less pathogenic cooperation with other connected microbial taxa in the rhizosphere soils. The structural equation model (SEM) revealed that pure and crude chitin amendments promoted soybean plant growth by indirectly regulating soil pH-mediated soil microbial activities and potentially beneficial microbes, respectively. Therefore, the reduction strategies for continuous cropping obstacles by adding pure and crude chitin were distinct; pure chitin amendments showed general disease suppression, while crude chitin exhibited specific disease suppression. Overall, chitin amendments could suppress potential plant pathogens and improve soil health, thereby promoting soybean growth, which provides new prospects for cultivation practices to control soybean continuous cropping obstacles.

## Introduction

Soybean (*Glycine max* L. Merill) is an important crop in China (Liu et al., 2008). The largest soybean production area in China is located in Heilongjiang Province, with the planting area increasing from 3.3 million ha in 2017 to nearly 6.8 million ha in 2022 (Yin et al., 2017; Wu, 2022). Given that soybean is highly susceptible to continuous cropping (Liu et al., 2019), an increasing frequency of soybean monoculture may result in extensive soil health deterioration and substantial losses of crop yield (Liu et al., 2012; Xu et al., 2021). Previous studies have documented that biotic factors, rather than abiotic factors, are the main reasons for the occurrence of continuous cropping obstacles of soybean (Dias et al., 2015). In addition, studies have also reported that yield reduction of soybean continuous cropping is mainly attributed to the depletion of beneficial microbes and the enrichment of pathogenic microbes and soybean cyst nematodes (Liu et al., 2020; Tian et al., 2020). Consequently, it is of great significance to monitor the changes in microbial communities in response to continuous cropping for soil ecological restoration and sustainable agricultural development.

Soil microorganisms play a pivotal role in plant growth, soil health and agroecosystem sustainability (Bonanomi et al., 2018; Lehmann et al., 2020) and participate in soil nutrient cycling and soil-borne disease development (Falkowski et al., 2008; Delgado-Baquerizo et al., 2016). The functional characteristics of soil microbial communities are key factors determining the emergence of crop soil-borne diseases (Xiong et al., 2020; Xun et al., 2021) and/or the formation of disease-suppressive soils (Liu et al., 2019; Liu et al., 2020). For example, potential plant pathogens *Fusarium* (Bai et al., 2015), *Clonostachys* (Bienapfl et al., 2012), *Lectera* (Liu et al., 2019) and *Pythium* (Bai et al., 2015) have been widely reported to cause soybean root rot and impair soybean growth. In contrast, potentially beneficial microbes, such as *Blastococcus* (Wang et al., 2020b), *Trichosporon* (Sláviková et al., 2002), *Bradyrhizobium* (Kalita and Malek, 2020) and *Rhizobacter* (Khan et al., 2015), can promote plant growth by participating in nitrogen fixation or organic matter degradation and increasing soil nutrient acquisition. Other species of potentially beneficial microbes, such as *Trichoderma* (Zhang et al., 2019), *Pseudomonas* (Zhou and Wu, 2018), *Beauveria* (Vega et al., 2008) and *Hirsutella* (Mwaheb et al., 2017), can protect plants from potential plant pathogens or soybean cyst nematode infestation by inducing systemic defense responses. Thus, monitoring these functional microbial groups associated with soil health and plant growth is crucial for revealing the mechanisms by which they are affected and the possible links between these effects and cropping systems.

Soil microorganisms live together to form a complex interaction system among species rather than living in isolation, and these interactions consequently influence microbial ecosystem functions and plant performance (Toju et al., 2018; Wagg et al., 2019). Co-occurrence patterns of microbial taxa are extremely important in understanding microbial community function by offering new insights into potential interaction and revealing niche spaces (Kara et al., 2013). Microbial network analysis has been proved to be a powerful tool for revealing the mechanisms associated with patterns of community assembly and elucidating the interactions among different taxonomic groups (Faust and Raes, 2012). Many studies have documented that the occurrence or suppression of soil diseases depends on complex biotic interactions between potential plant pathogens and beneficial microbes (Saleem et al., 2017; Durán et al., 2018; Li et al., 2021b). A typical example of the complex interactions between plants and microbiomes is the effect of disease-suppressive soils or conducive soils induced by different agronomic practices (Xiong et al., 2017). It was reported that the application of amendments, such as organic fertilizer (Chen et al., 2022), lime (Li et al., 2022), biochar (Yao et al., 2017) and chitin (Inderbitzin et al., 2018), effectively ameliorated soil acidification by reshaping the microbial communities. However, understanding of if and how microbial communities support the beneficial effects of such amendments on the prevention of soil-borne diseases in continuous cropping systems remains unclear.

Given the adverse effects of soybean continuous cropping on soil quality and soybean growth, various strategies have been employed to overcome soybean continuous cropping obstacles. However, traditional control practices for continuous cropping obstacles, including the use of crop rotation (Li et al., 2021a) and chemical agents (Pu et al., 2022), still have potential drawbacks. Crop rotation requires long periods to reduce soil-borne pathogens and eliminate the negative effects of continuous cropping (Ding et al., 2021; Li et al., 2021a), and its effectiveness is limited once the disease breaks out (Wang et al., 2013). Chemical fumigants and fungicides have high efficiency in killing soil microorganisms (Fang et al., 2019), but the intensive use of chemicals not only causes severe environmental pollution (Liu et al., 2015) but also encourages the development of target pathogen resistance (Goldman et al., 1994). Organic amendments are known to be attractive alternatives to relieve continuous cropping obstacles (Zhou et al., 2021). Chitin, as the most abundant polysaccharide in nature, possesses good biocompatibility, biodegradability, nontoxicity and various biological activities and poses no hazard to the environment (Zhan et al., 2021; Kemboi et al., 2022). More importantly, chitin also has great potential for improving soil quality, inducing plant disease resistance and promoting plant growth (Fan et al., 2020b; Shamshina et al., 2020). Chitin amendments have been reported to promote plant growth by activating chitinolytic microbes and releasing nitrogen and also to enhance plant disease resistance (De Tender et al., 2019). Consequently, chitin amendments could be a promising and environmentally friendly control strategy for eliminating soybean continuous cropping obstacles (Kielak et al., 2013; Debode et al., 2016). However, studies on how the microbial community regulated by chitin amendments influences both soil health and plant growth are still very limited.

In this study, we established a pot experiment by adding pure and crude chitin with different dosage gradients into a continuous cropping soil. We investigated the bidirectional effects of chitin amendments on bacterial and fungal communities and soil microbe–plant interactions in both the bulk and rhizosphere soils using high-throughput sequencing technology. We mainly investigated (i) the variations in soil chemical properties and soil microbial activities in responses to chitin amendments; (ii) the relative abundances of potential beneficial microbes and plant pathogens and their interactions induced by chitin amendments; (iii) the impacts of soil properties resulting from chitin amendments on soil microbial activities, potential beneficial microbes and plant pathogens, finally on soybean growth. Three hypotheses were tested in the present study: (i) chitin amendments would increase soil pH and available nutrient contents; (ii) chitin amendments may induce looser pathogenic subnetwork structures and less pathogenic cooperation; (iii) the variations in soil chemical properties would induce higher soil microbial activities and lower relative abundances of potential plant pathogens, promoting soybean growth.

## Materials and methods

### Study site description and pot experimental design

The pot experiment was established on 10 May 2019 at the experimental garden of the Northeast Institute of Geography and Agroecology, Chinese Academy of Sciences, Harbin, Heilongjiang Province, China (45°41′48″N, 126°38′12″E). The soils used in the pot experiments were 0-20 cm topsoil collected from fields with continuous soybean cropping for five years in Glory Village, Hailun, Heilongjiang Province, China (47°21′N, 126°50′E). The exogenous chitin of pure chitin (PC) and crude chitin (CC) were purchased from Jinan Apollo Crustacean Fertilizer Co., Ltd. and Shanghai Haike Shengwu Biotech Co., Ltd., respectively, and were used to assess the effectiveness of exogenous chitin on continuous cropping obstacles. Crude chitin is a biopolymer (Debode et al., 2016; Zhan et al., 2021) that is mainly sourced from the shells of marine organisms, such as shrimp, lobsters and crabs, while pure chitin is purified from crude chitin by removing calcium and protein with acid and alkali (Inderbitzin et al., 2018; Kemboi et al., 2022). The total carbon and nitrogen contents were 187.7 g/kg and 31.5 g/kg in pure chitin and 396.3 g/kg and 72.4 g/kg in crude chitin, respectively.

Seven treatments with different chitin addition ratios were designed in this study. The addition ratios of pure chitin were 0.05%, 0.1% and 0.2%, while those of crude chitin were 0.5%, 1% and 2%, which were calculated based on the soil mass ratios used in each pot. These treatments were marked as PC1, PC2, PC3, CC1, CC2, and CC3 for pure chitin and crude chitin, respectively. The no-addition treatment was designed as a control and marked as C0. Each treatment was set with eight replicates (pots), and 3 kg of soil was placed in each pot, whose height and diameter were 17.5 cm and 11.0 cm, respectively. The added chitin was thoroughly mixed with the soil before soybean planting. Six soybean seeds were sown in each pot, while four soybean seedlings were kept in each pot at the stage of cotyledon extension. Subsequently, soybean plants were watered with the same amount of water every two days to maintain a relatively consistent water retention capacity.

In total, 56 bulk and 56 rhizosphere soil samples were collected (seven treatments × eight replicates) when soybeans reached the flowering stage in 2019. A single bulk soil sample (replicate) was collected from each pot and then sieved through a 2 mm mesh to fully homogenize and remove impurities. Bulk soils were placed into a sterile 50 mL centrifuge tube, and the remaining soil was put in a reclosable bag. Rhizosphere soil samples were collected by shaking methods to preserve soil firmly attached to the root surface (Fan et al., 2020a). A portion of each collected rhizosphere soil sample was placed in a sterilized 2 mL centrifuge tube. The centrifuge tubes were kept at -80°C for subsequent molecular analysis, while the rest of the soils and soybean plants were temporarily kept in a 4°C refrigerator prior to downstream determination for the determination of the physicochemical properties and soybean plant morphological characteristics.

### Measurement and analysis of soil chemical properties

Considering that the quantity of collected rhizosphere soils was too small to determine the chemical properties, soil chemical properties were measured only in bulk soils. Soil pH was determined using a pH meter (Thermo Electron, USA) from a soil water supernatant at a 1:5 (weight/volume) ratio of soil to 0.01 mol L^-1^ CaCl_2_ solution after shaking for 17 hours (Wang et al., 2015). The water used to prepare the CaCl_2_ solution was carbon dioxide free to minimize the impact of carbon dioxide on soil pH. Soil total carbon (TC) and nitrogen (TN) were assayed by the combustion method (Fan et al., 2019) using a CNS Elemental analyzer (VarioEL III, Germany) after air-dried soils passed through 0.25 mm sieves. After the soil samples were melted by sodium hydroxide, the soil total phosphorus (TP) and potassium (TK) contents were determined with the molybdenum blue method and flame spectrophotometry method (FP640, INASA Instrument, China), respectively. Ammonium ( ${\text{NH}}_{4}^{+}$ -N) and nitrate nitrogen ( ${\text{NO}}_{3}^{-}$ -N) were extracted via 2 mol L^-1^ KCl solution at a 1:10 (weight/volume) ratio of fresh soil to KCl solution. The soil ammonia nitrogen ( ${\text{NH}}_{4}^{+}$ -N) and nitrate nitrogen ( ${\text{NO}}_{3}^{-}$ -N) contents were analyzed from the supernatant using a continuous flow analytical system (SKALAR SAN++, Skalar, Netherlands). After extraction with 0.5 mol L^-1^ NaHCO_3_ and 1.0 mol L^-1^ CH_3_COONH_4_ solution, soil available phosphorus (AP) and potassium (AK) contents were quantified with molybdenum blue and flame photometry methods (FP640, INASA Instrument, China), respectively.

### Soybean plant morphological characteristics and soil total microbial activity measurement

Plant height was determined with a tapeline to measure the length from the soybean cotyledon scar to the top growth point of the main stem. We cut the plants from the cotyledon scar and weighed them to obtain the plant fresh weight. Root length, root surface area and root volume were measured using a root analyzer (WinRHIZO, Canada) after root nodules were completely removed with tweezers. The fresh soybean plants and roots were enwrapped into envelopes, desiccated at 105°C for 30 minutes to degreen, switched to 80°C for 48 hours, and finally weighted to measure plant and root dry weight.

The total microbial activity of bulk soil was determined by the fluorescein diacetate (FDA) hydrolysis method (Gillian and Harry, 2001). First, 15 mL potassium phosphate buffer (60 mmol L^-1^; pH=7.6) and 0.2 mL FDA stock solution (1000 μg mL^-1^) were added to 2.0 g fresh soil to initiate the hydrolysis reaction. Then, after incubation at 30 °C for 20 minutes, 15 mL chloroform/methanol (2:1; v/v) was added to terminate the hydrolysis reaction. Finally, the absorbance of the supernatant was determined with a spectrophotometer (PERSEE T6-1650E) at a wavelength of 490 nm, and the fluorescein content was calculated according to the standard curve to obtain the soil total microbial activity.

### DNA extraction, PCR amplification and high-throughput sequencing

The soil microbial DNA for each sample was extracted from 0.5 g fresh soil (stored in a -80 °C freezer) using the Fast DNA^®^ Spin Kit for Soil (MP Biomedicals, Santa Ana, CA, USA) according to the manufacturer's manual. The extracted DNA was measured for concentration and purity with a NanoDrop 2000 spectrophotometer (Thermo Scientific, Wilmington, DE, USA), and the quality of extracted DNA was examined using 1% agarose gel electrophoresis.

The V3-V4 region of the bacterial gene and the ITS region of the fungal gene were amplified by the primer pairs 515F (5’-GTG CCA GCM GCC GCG GTA A-3’)/907R (5’-CCG TCA ATT CCT TTG AGT TT-3’) (Walters et al., 2016) and ITS1F (5’-CTT GGT CAT TTA GAG GAA GTA A-3’)/ITS2R (5’-GCT GCG TTC TTC ATC GAT GC-3’) (Adams et al., 2013), respectively, with the forward primer labeled with a sample-specific barcode sequence (6 bp) at the 5’ end to differentiate the amplified products. PCR amplification was conducted in a 25 μL reaction system comprising 2.5 μL dNTPs (2.5 mM), 2.5 μL 5 × FastPfu Buffer, 0.2 μL BSA, 0.5 μL FastPfu Polymerase, 0.25 μL forward and reverse primers (5 μM), 1.0 μL template DNA (10 ng) and 17.8 μL sterilized ultrapure water (ddH_2_O). The PCR amplification procedure was performed as reported in the literature (Adams et al., 2013; Walters et al., 2016). Each sample was amplified in triplicate, and the PCR products were combined and purified using an agarose gel DNA purification kit (TaKaRa, Dalian, China), examined in a 2% agarose gel and quantitated by a QuantiFluor™-ST Fluorometer (Promega, Madison, WI, USA) following the manufacturer’s protocols. The equimolar amounts of purified PCR amplicons were combined as one sample and paired-end sequenced (2 × 300 bp) using the Illumina MiSeq PE 300 platform at Majorbio Bio-Pharm Technology Co., Ltd. (Shanghai, China). The raw sequencing data of bacteria and fungi from this research were stored in the NCBI Sequence Read Archive (SRA), with accession numbers PRJNA869919 and PRJNA870067, respectively.

### Sequencing data analysis

The raw data from sequencing were analyzed using the QIIME-1.9.1 (Quantitative Insights Into Microbial Ecology) pipeline (http://qiime.sourceforge.net/) (Caporaso et al., 2010) and merged through FLASH-1.2.7 (fast length adjustment of short reads) (http://ccb.jhu.edu/software/FLASH/) software (Magoč and Salzberg, 2011). First, we acquired 3,615,418 (from 18,302 to 46,619) and 7,120,035 (from 29,755 to 93,365) high-quality sequences of bacteria and fungi across soil samples after eliminating low-quality sequences (average base quality score< 20 reads and/or length< 200 bp) (Aronesty, 2011), mismatching the primers and barcodes (Fan et al., 2019) and filtering the chimeras with the UCHIME algorithm (Edgar, 2010). Then, the high-quality sequences were clustered into operational taxonomic units (OTUs) based on a 97% similarity threshold through UPARSE software (http://drive5.com/uparse/) (Edgar, 2013). The taxonomic identity of OTUs was executed through the RDP (Ribosomal Database Project) classifier (version 11.5; Cole et al., 2014) at the 80% confidence threshold based on the SILVA (http://www.arb-silva.de/) (release 132; Quast et al., 2013) and UNITE (https://unite.ut.ee/) (version 8.0; Abarenkov et al., 2010) databases for bacteria and fungi, respectively. The sequences that mismatched with bacteria and fungi were eliminated from the dataset after taxonomic identity. Furthermore, the potential plant pathogens were classified using FUNGuild (https://github.com/UMNFuN/FUNGuild) (Nguyen et al., 2016). All taxonomic information for the fungal nodes in each community network was uploaded to the online application in FUNGuild, and functional group (also referred to as guild) assignments of nodes that matched at a ≥ 93% were accepted (Nilsson et al., 2008). The fungal taxa were identified as plant pathogen with their confidence rankings of “Highly Probable” and “Probable” in current study (Liu et al., 2020; Hu et al., 2021). In addition, potentially beneficial bacteria and fungi with various ecological functions were reconfirmed in the published literature (**Table S1**). Considering the impact of sequence number variation among samples, based on the minimum sequence, a subset of 18,302 and 29,755 sequences for each sample of bacteria and fungi were randomly selected for subsequent analysis.

### Ecological network construction and analysis

To uncover the interactions of bacterial and fungal taxa, co-occurrence network analysis, based on random matrix theory (RMT) methods, was performed using OTU relative abundance data through the Molecular Ecological Network Analysis Pipeline (MENAP) (http://129.15.40.240/mena/) (Zhou et al., 2011). We filtered out the rare OTUs with relative abundances< 0.01% and merged the remaining bacterial and fungal OTUs of each treatment into an abundance table. Eight replicates of each treatment for bulk and rhizosphere soil samples were uploaded to the pipeline, and only OTUs observed in 6 out of 8 replicates were used to construct networks. The network construction procedures were performed as follows. First, after uploading the OTU file into the MENA pipeline, the proper similarity threshold (St) (*p*<0.01) was automatically generated based on the RMT method. An edge or link between OTU pairs was assigned when the correlation exceeded *St* through Pearson’s correlation analysis. Second, the calculations of “global network properties”, “individual nodes’ centrality” and “module separation and modularity” were conducted through the pipeline. Third, the network attribute files were generated by running the “output for the Cytoscape software visualization” based on the “greedy modularity optimization” algorithm (Clauset et al., 2004), and then the co-occurrence networks were visualized with Cytoscape-3.7.2 (https://cytoscape.org/) (Shannon et al., 2003). Finally, random networks, with identical nodes and edges as the empirical networks, were generated by running the “randomize the network structure and then calculate network properties”. Subsequently, the topological indices of the empirical and random networks were compared to identify if the network properties were error prone. In addition, pathogen-associated subnetworks were extracted from the global networks using the tool ‘First Neighbors of Selected Nodes’ in Cytoscape to explore the effects of chitin amendments on the cooccurrence patterns of potential plant pathogens.

### Structural equation modeling construction and analysis

Structural equation modeling (SEM) was constructed to assess the direct and indirect influences of the prominent soil chemical properties on beneficial microbial taxa, potential plant pathogens, FDA (fluorescein diacetate) and plant growth performance (Grace, 2006). The SEM analyses were carried out using IBM SPSS Amos-21.0 software (Chicago, IL: Amos Development Corporation). The soil physicochemical variables included soil pH, TN, AP and TK, which were preselected by the Mantel test and autocorrelation test using R software (Version 3.6.2) and IBM SPSS-20.0 software, respectively. All variables used in SEM were mean standardized by Z transformation (mean=0, standard deviation=1) using the “scale” function in R-3.6.2 to improve normality of sample data (Zhao et al., 2019). The maximum likelihood estimation method was applied to fit the covariance matrix of these variables into the model. The path represented the standardized partial correlation coefficient, which illustrated the magnitude of the relationship between two factors. Latent variables, such as soybean plant morphological characteristics, were employed to integrate the effects of multiple conceptually related observed variables into a single composite effect, which could help to better interpret the model results (Delgado-Baquerizo et al., 2020).

### Statistical analyses

A subset of 18,302 and 29,755 sequences were randomly selected for each bacterial and fungal sample, respectively, to analyze the microbial alpha-diversity and beta-diversity, with a view to comparing the relative differences among soil samples. The number of OTUs and Faith’s PD (phylogenetic distance) (Faith, 1992) were used to compare soil bacterial and fungal alpha diversity using the QIIME platform (http://qiime.org/index.html) with the alpha diversity.py function. Significance differences of bacterial and fungal alpha diversity between pure and crude chitin treatments was analysed by t-test. One-way ANOVA (analysis of variance), based on Duncan’s post-hoc test, was employed to assess the differences in the soil physicochemical properties, bacterial and fungal relative abundance, alpha diversity indices and plant growth indices, which was implemented in SPSS-20.0 software. The variations in the bacterial and fungal community composition and relative abundance at the phylum level among different treatments were illustrated by stacked bar charts, which were generated using the “ggplot2” package in the R platform (R Development Core Team, 2016). A column chart was constructed to depict the significant differences in the relative abundance of potentially pathogenic and beneficial microbes with an average relative abundance > 0.1% in each treatment at the OTU level. In addition, NMDS (nonmetric multidimensional scaling) and Adonis (nonparametric multivariate analysis of variance) analyses based on the Bray–Curtis dissimilarity matrix were carried out using the “vegan” R package to explore the significant differences in bacterial and fungal community structure across different bulk and rhizosphere soil samples (Oksanen et al., 2022). Permutational multivariate analysis of variance (PERMANOVA) was performed to assess the effects of the compartment and amendment type on the community composition of bacterial and fungal groups using the adonis function with 999 permutations in the “vegan” package in R (version 3.4.3) (Guo et al., 2022). Redundancy (RDA) analyses were performed to further identify the possible correlations between bacterial and fungal community structures and environmental variables. The environmental variables used in the RDA and SEM analysis were preselected by the Mantel test and variance inflation factor (VIF). The RDA, Mantel test and VIF analysis mentioned above were all conducted with the “vegan” package in R (version 3.6.2), and the significant differences were examined with the “envfit” function with 999 permutations (Guo et al., 2021). Meanwhile, the random forest model was employed to evaluate the importance of environmental variables in predicting the changes in bacterial and fungal community structures using the “rfPermute” package in the R environment (Archer, 2020).

## Results

### Changes in soil chemical properties and plant morphological characteristics

Chitin amendments significantly improved soil chemical properties compared to that in the control. Briefly, soil pH, TK, AP and AK were markedly increased in PC and CC amendments, except for TK in PC1B and AK in PC2B and CC1B (**Table 1**). CC remarkably elevated the soil TN in CC2B and CC3B, but the soil TC, C/N ratio and ${\text{NH}}_{4}^{+}$ -N content were significantly reduced by the PC and CC amendments, with the exception of TC in CC3B (*P*< 0.05) (**Table 1**). In addition, the chitin amendments sharply decreased the soil nitrate nitrogen ( ${\text{NO}}_{3}^{-}$ -N) (*P*< 0.05), while it was highly increased in CC2B and CC3B (*P*< 0.05) (**Table 1**).

**Table 1:** Soil chemical properties under different pure and crude chitin addition dosages in the bulk soils.

Treatment	pH	Total C(g/kg)	Total N(g/kg)	Total P(g/kg)	Total K(g/kg)	${\text{NH}}_{4}^{+}$ -N (mg/kg)	${\text{NO}}_{3}^{-}$ -N (mg/kg)	Available P(mg/kg)	Available K (mg/kg)
C0B	5.10±0.02e	18.77±0.20a	1.61±0.04cd	0.53±0.08ab	15.45±0.11b	63.70±7.52a	13.56±1.02c	23.54±2.50d	139.5±2.7d
PC1B	5.77±0.03d	17.57±0.27b	1.62±0.04cd	0.50±0.08b	15.83±0.62b	48.88±2.83c	1.55±0.34d	27.56±3.77bc	162.3±7.4b
PC2B	5.71±0.08d	17.26±0.51b	1.56±0.06d	0.61±0.05a	17.06±0.75a	51.25±3.27c	1.76±0.61d	26.22±1.40c	143.3±4.7d
PC3B	5.78±0.06d	17.54±0.73b	1.60±0.10cd	0.56±0.08ab	17.03±0.22a	51.49±2.24c	1.99±0.47d	28.74±1.70abc	152.1±6.8c
CC1B	6.34±0.03c	17.42±0.47b	1.69±0.06c	0.60±0.12a	16.82±0.39a	42.73±2.30d	1.81±0.38d	30.09±2.55ab	144.1±13.7d
CC2B	6.83±0.05b	17.81±0.13b	1.77±0.06b	0.48±0.10b	16.82±0.35a	44.18±3.59d	23.20±2.95b	30.57±1.76a	153.2±2.5c
CC2B	6.97±0.13a	18.69±0.43a	2.00±0.12a	0.51±0.02b	16.63±0.73a	57.73±6.34b	47.07±5.04a	27.60±1.94bc	182.7±9.7a

C0, PC1, PC2, PC3 and CC1, CC2, CC3 represent the pure and crude chitin amendment rate at 0%, 0.05%, 0.1%, 0.2% and 0.5%, 1%, 2% of total soil mass, respectively. B: bulk soil. Means of 8 replicates are presented with standard deviations. Different letters within the same column show significant differences among treatments by one-way ANOVA (P< 0.05).

Compared with C0B, soybean plant morphological characteristics, such as plant height, root dry weight, and aboveground dry and fresh weight, were significantly increased under PC and CC amendments (**Figures S1A-G**). In addition, with the exception of PC2B and PC3B, soybean root volume was significantly (*P*< 0.05) increased in both the PC and CC amendments (**Figure S1H**). However, there were no significant differences in such plant morphological characteristics among the different pure chitin addition dosages (*P*< 0.05) (**Figures S1B, D-H**).

### Distribution patterns of bacterial and fungal communities

Compared with C0B, the bulk soil microbial activity was significantly increased under chitin amendments (*P*< 0.05) (**Figure S1E**). In the bulk soils, except for PC1B, both PC and CC had a significantly (*P*< 0.05) lower bacterial and fungal alpha diversity than C0B (**Figures S2A, B**). In contrast, the alpha diversity of the bacterial and fungal communities in the rhizosphere soils was hardly affected by PC (*P* > 0.05), whereas it was markedly (*P*< 0.05) decreased in the CC treatments, except for fungal alpha diversity in PC1B (**Figures S2C, D**). In addition, the alpha diversity of bacterial and fungal communities was dramatically lower in CC than in PC in both bulk and rhizosphere soils (*P*< 0.05) (**Figure S2**, **Table S2**).

**Figure 1:**
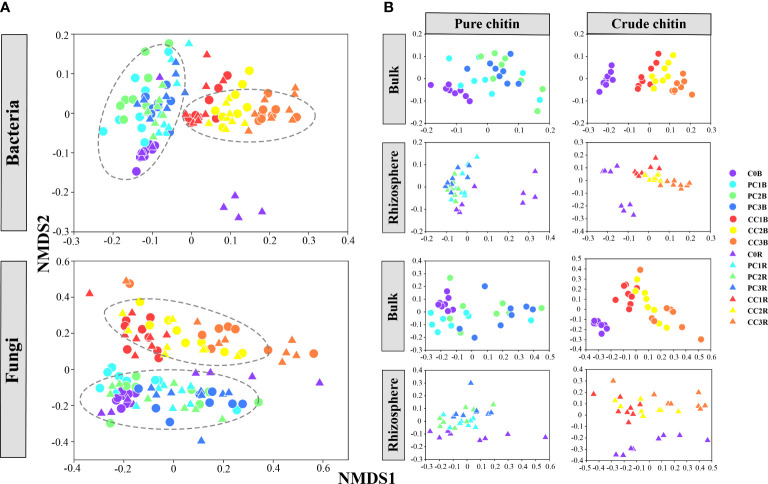
Nonmetric multidimensional scaling (NMDS) plot of all soil bacterial and fungal communities **(A)**. The separated NMDS plots showed the effects of pure and crude chitin amendments on the bacterial and fungal community structures in the bulk and rhizosphere soils, respectively **(B)**. C0, PC1, PC2, PC3 and CC1, CC2, CC3 represent the pure and crude chitin amendment rate at 0%, 0.05%, 0.1%, 0.2% and 0.5%, 1%, 2% of total soil mass, respectively. B, bulk soil; R, rhizosphere soil.

The beta diversity of microbial communities illustrated by NMDS plots clearly showed that the bacterial and fungal communities were obviously divided into two major groups, that is, different kinds of chitin amendments (PERMANOVA, *P<* 0.05) (**Figure 1A**, **Table S3**), whereas the microbial communities in the bulk and rhizosphere soils were grouped closely (**Figure 1A**). In addition, the separated NMDS plots showed a clear separation between the different dosages of pure and crude chitin additions, which also differed from the control (Adonis tests, *P<* 0.05) (**Figure 1B**, **Table S4**). Mantel test analysis implied that soil pH, TC, TN, C/N ratio, TK, AP, AK, ${\text{NH}}_{4}^{+}$ -N, and ${\text{NO}}_{3}^{-}$ -N were significantly correlated with the bacterial and fungal community structures in the bulk soils (*P*< 0.05) (**Table S5**). Based on Mantel test results and VIF screening, RDA revealed that soil pH, TN and ${\text{NO}}_{3}^{-}$ -N made the greatest contributions to the variations in both bacterial and fungal community structures (*P* = 0.001) (**Figures S3A, C**, **Table S6**). Moreover, these observations were supported by the results of random forest (RF) analysis (**Figure S3B, D**).

### Changes in dominant bacterial and fungal taxa

**Figure 2:**
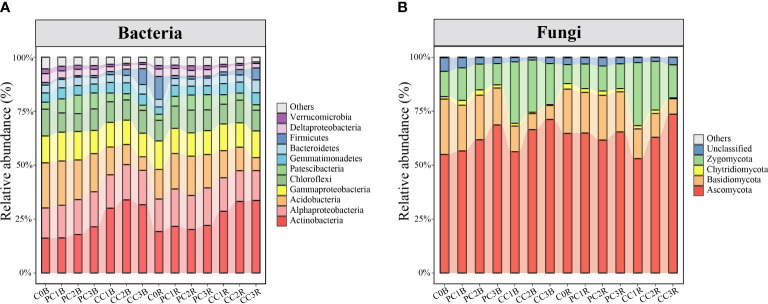
Relative abundance of the dominant bacterial and fungal phyla/classes (at least one treatment > 1%) **(A, B)**. The treatment description is same as **Figure 1**.

In total, 6,012,813 good-quality bacterial sequences (32,046-74,775 per sample; mean = 53,658) and 7,256,335 good-quality fungal sequences (30,599-94,360 per sample; mean = 64,788) were obtained from 112 soil samples in this study. When grouped at the 97% similarity threshold, these sequences were clustered into 9,113 and 2,611 OTUs for bacteria and fungi, which were identified to 36 and 8 phyla, respectively. The bacterial and fungal community compositions in the bulk and rhizosphere soils under different PC and CC amendments are shown in **Figure 2**.

**Figure 3:**
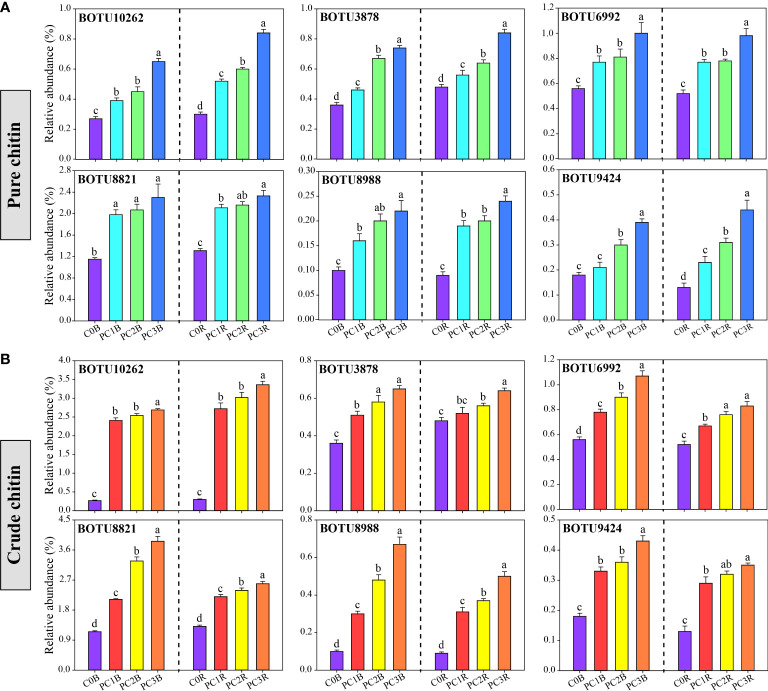
Relative abundance of potentially beneficial bacteria under different pure **(A)** and crude **(B)** chitin addition dosages. The bars indicate the means of 8 replicates and the error bars represent the standard deviation. Different letters indicate significant differences among treatments (one-way ANOVA, *P*< 0.05, Duncan’s multiple-range test). The treatment description is same as **Figure 1**.

At the bacterial phylum level, the PC markedly increased and decreased the relative abundances of Patescibacteria and Firmicutes in the bulk and rhizosphere soils, respectively, whereas it decreased the relative abundances of Deltaproteobacteria and Chloroflexi in the bulk soils and Gammaproteobacteria in the rhizosphere soils (one-way ANOVA, *P*< 0.05) (**Figure 2A**, **Table S7**). In contrast, CC markedly reduced the relative abundances of Deltaproteobacteria, Acidobacteria and Chloroflexi in both bulk and rhizosphere soils, while remarkably increased the relative abundances of Actinobacteria and Gemmatimonadetes in both bulk and rhizosphere soils, and increased Alphaproteobacteria in the bulk soils, except for CC1B (*P*< 0.05) (**Figure 2A**, **Table S8**). Meanwhile, the relative abundance of Actinobacteria was significantly increased by a high level of PC, and that of Bacteroidetes was promoted by a high level of CC in both bulk and rhizosphere soils (*P*< 0.05) (**Figure 2A**, **Tables S7**, **S8**). At the bacterial OTU level, chitin amendments, especially CC, distinctly increased the relative abundance of OTUs in both bulk and rhizosphere soils, which belonged to *Streptomyces*, *Sphingomonas*, *Bacillus*, *Mesorhizobium* and *Nitrospira* (**Figures 3A, B**, **Tables S9**, **S10**).

**Figure 4:**
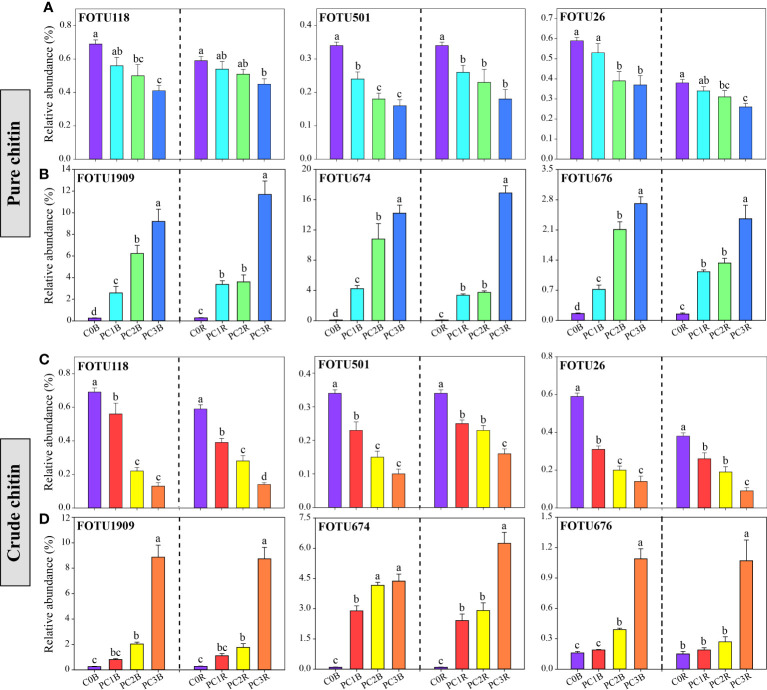
Relative abundance of potentially pathogenic **(A, C)** and beneficial **(B, D)** fungi under different pure and crude chitin addition dosages. The bars indicate the means of 8 replicates and the error bars represent the standard deviation. Different letters indicate significant differences among treatments (one-way ANOVA, *P<* 0.05, Duncan’s multiple-range test). The treatment description is same as **Figure 1**.

At the fungal phylum level, the relative abundance of Basidiomycota was dramatically decreased by chitin amendments, but that of Zygomycota was markedly increased by CC in both bulk and rhizosphere soils (*P*< 0.05) (**Figure 2B**, **Tables S7**, **S8**). The high level of PC markedly increased and reduced the relative abundances of Ascomycota and Zygomycota in the bulk soils, respectively, while the high level of CC significantly promoted the relative abundance of Ascomycota in both bulk and rhizosphere soils (*P*< 0.05) (**Figure 2B**, **Tables S7**, **S8**). At the fungal OTU level, two high levels of PC and CC significantly increased the abundance of the biocontrol fungi *Purpureocillium* and *Penicillium* (**Figures 4B, D**, **Tables S9**, **S10**). Additionally, PC amendments also remarkably increased the relative abundance of the fungal parasites FOTU2062 (*Metarhizium frigidum*) and FOTU843 (*Pochonia chlamydosporia*), while CC amendments increased the abundance of *Mortierella* (**Tables S9**, **S10**). However, CC and high levels of PC dramatically reduced the relative abundance of potential pathogens affiliated with *Fusarium*, *Paraphoma*, *Cylindrocarpon* and *Septoria* (**Figures 4A, C**, **Tables S9**, **S10**).

### Cooccurrence patterns of bacterial and fungal networks

**Figure 5:**
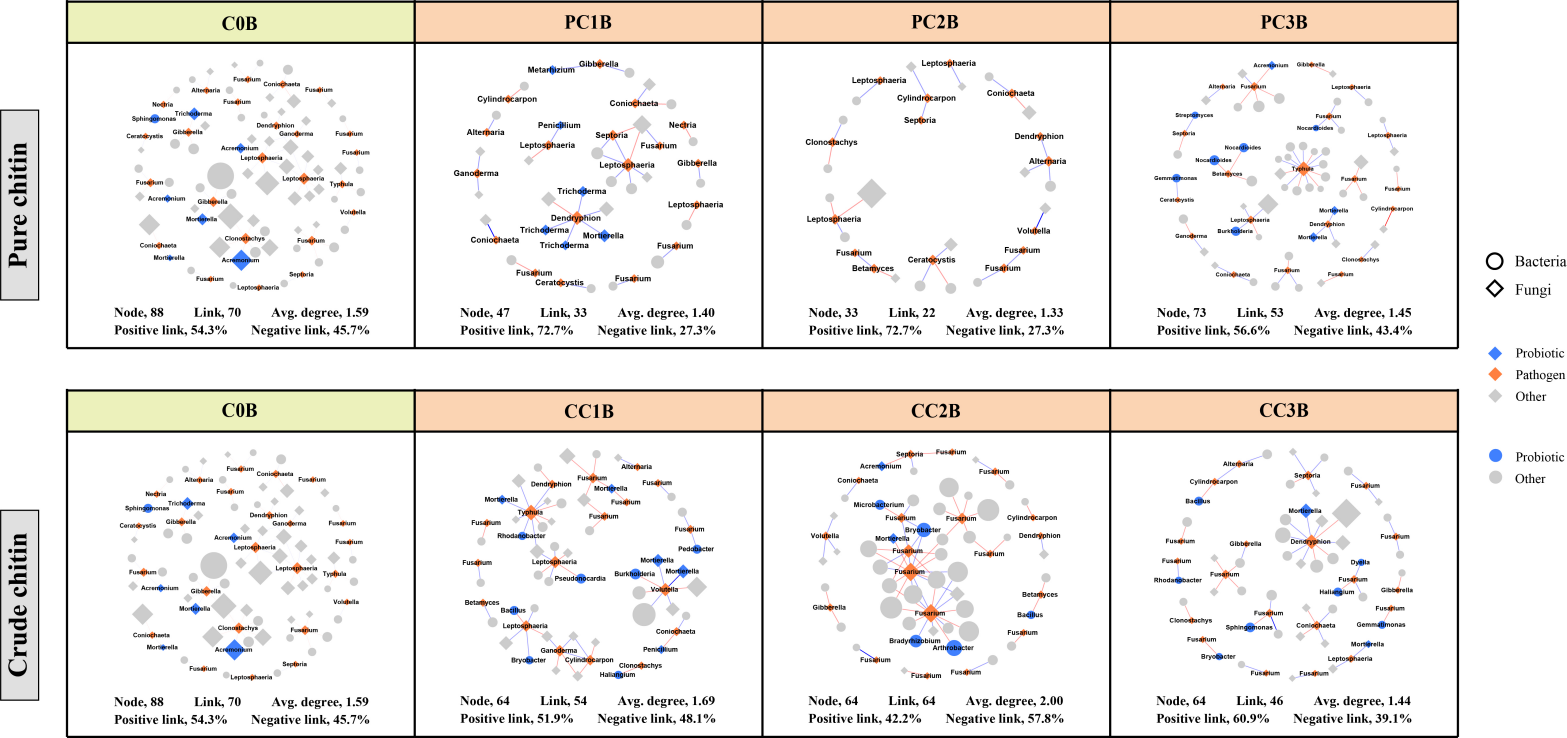
Subnetworks of potential plant pathogens separated from the microbial network under different pure and crude chitin addition dosages in the bulk (B) soils. Circles and diamonds represent bacteria and fungi, respectively. The size of each node is proportional to degree. Node with blue, orange and grey indicate probiotic, pathogen and other, respectively. The microbial taxa are labeled in the blue and orange nodes. Blue and red lines show positive and negative interactions between two individual nodes, respectively. The treatment description is same as **Figure 1**.

**Figure 6:**
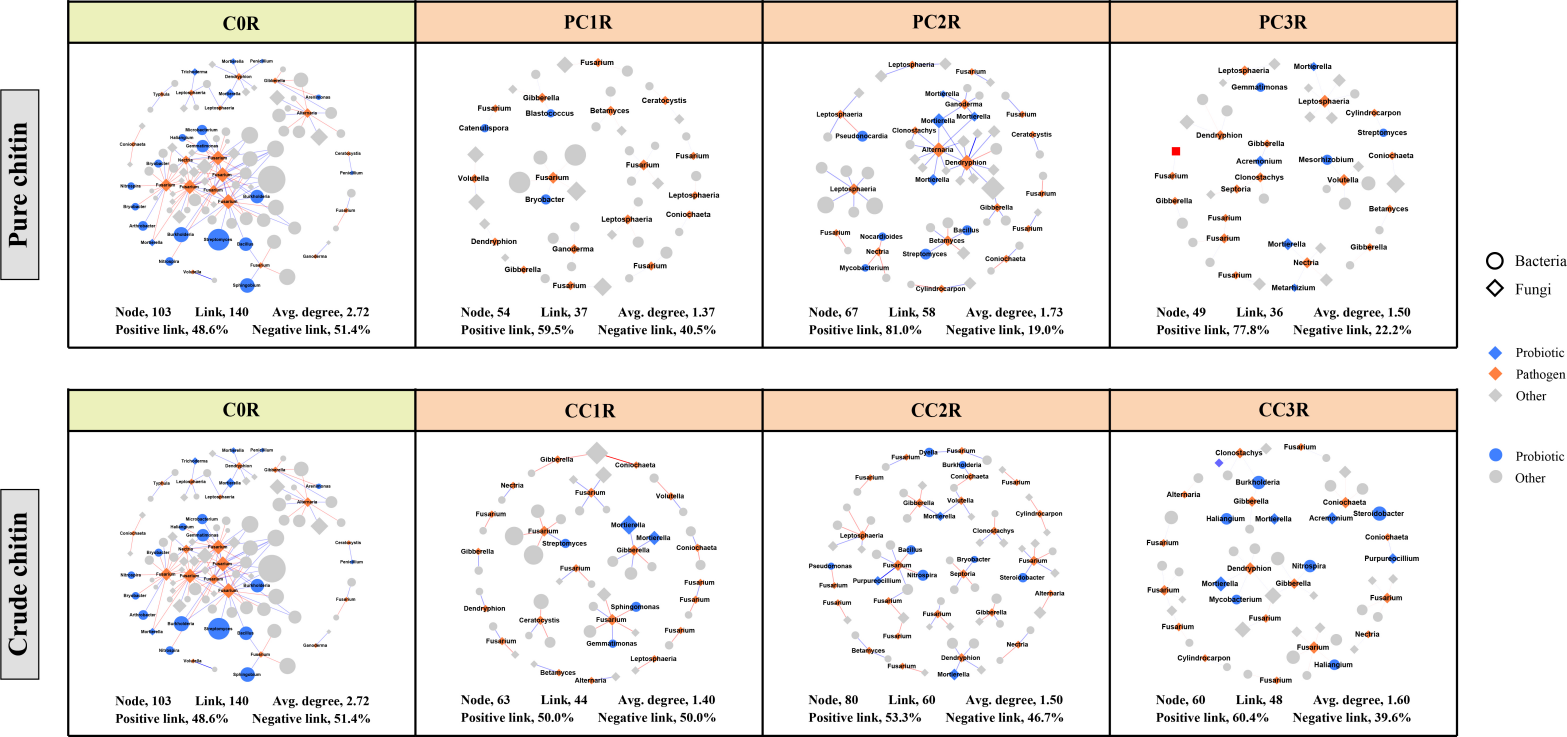
Subnetworks of potential plant pathogens separated from the microbial network under different pure and crude chitin addition dosages in the rhizosphere (R) soils. Circles and diamonds represent bacteria and fungi, respectively. The size of each node is proportional to degree. Node with blue, orange and grey indicate probiotic, pathogen and other, respectively. The microbial taxa are labeled in the blue and orange nodes. Blue and red lines show positive and negative interactions between two individual nodes, respectively. The treatment description is same as **Figure 1**.

To assess the effects of chitin amendments on soil microbial interactions, seven cooccurrence networks integrating bacterial and fungal OTUs were generated for different dosages of chitin in the bulk and rhizosphere soils, respectively (**Figures S4**, **S5**). The integrated network degrees for bacterial and fungal nodes followed a power-law distribution in both bulk and rhizosphere soils (*R^2^* > 0.6) (**Table S11**), indicating a nonrandom cooccurrence distribution pattern. The corresponding subnetworks of potential plant pathogens were further assessed from each integrated microbial community global network (**Figures 5**, **6**). In the rhizosphere soils, we found that chitin amendments could cause simpler pathogenic-associated networks with apparently lower average node degrees in the PC and CC subnetworks than in C0B (**Figure 6**). Furthermore, the direct interactions between potential plant pathogens and other microbial taxa are shown in **Table S12**. Compared to the C0B subnetwork, negative interactions between *Bacillus* and the potential plant pathogens *Cylindrocarpon* and *Fusarium* were detected in subnetworks of CC treatment in the bulk and rhizosphere soils, respectively (**Table S12**). Additionally, a negative interaction between *Fusarium* and *Sphingomonas* was observed in the subnetwork of CC amendment (**Table S12**). The potential plant pathogen *Cylindrocarpon* negatively interacted with *Nocardioides* and *Mycobacterium* in the CC2R subnetwork (**Table S12**). Intriguingly, a negative interaction between *Fusarium* and *Mortierella* was observed in pure and crude chitin subnetworks in the bulk soils, but it showed different trends in the rhizosphere soils (**Table S12**).

### Abiotic and biotic factors influencing soybean growth

**Figure 7:**
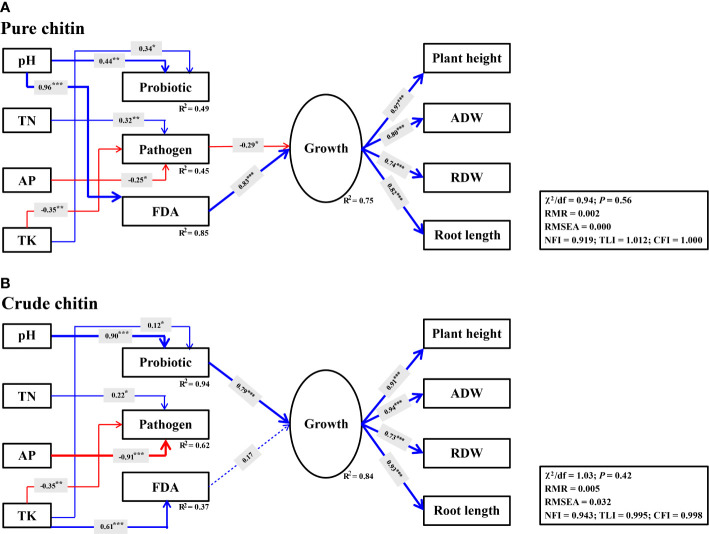
Structural equation models (SEMs) describing the direct and indirect effects of soil properties, probiotic, potential plant pathogens and FDA on soybean growth under pure **(A)** and crude **(B)** chitin amendments. The probiotic represent potentially beneficial bacteria and fungi. The latent variable for soybean growth are indicated by plant height, aboveground dry weight (ADW), root dry weight (RDW) and root length. Solid and dashed lines indicate significant and insignificant relationships, respectively. Arrow width indicates the effect strength, with blue and red indicating positive and negative effects, respectively. *R^2^* means the proportion of variance explained. ***P< 0.001; **P< 0.01; *P< 0.05.

SEM was used to evaluate whether the changes in soil properties induced by soil amendments directly and/or indirectly affected potential plant pathogens and beneficial species, which ultimately affected soybean growth. It was observed that soil variables and microbial parameters explained higher variations in soybean plant growth under crude chitin application (*R^2^* = 0.84) than under pure chitin application (*R^2^* = 0.75). With respect to pure chitin application, soil properties jointly explained 45% (*R^2^* = 0.45) of the variation in potential plant pathogens, among which TN (*γ* = 0.32, *P*< 0.01) was directly positively associated with potential plant pathogens, while AP (*γ* = -0.25, *P*< 0.05) and TK (*γ* = -0.35, *P*< 0.01) were negatively associated with potential plant pathogens and thus indirectly and negatively affected soybean plant growth (*γ* = -0.29, *P*< 0.05) (**Figure 7A**). Importantly, soil pH (*γ* = 0.96, *P*< 0.001) was significantly and positively correlated with soil microbial activity (FDA) and then exhibited significant indirect and positive effects on soybean plant growth (*γ* = 0.83, *P*< 0.001) (**Figure 7A**). Regarding crude chitin application, soil TK (*γ* = 0.61, *P*< 0.001) had significant and direct positive correlations with soil microbial activity (FDA) but exerted no significant indirect positive effects on soybean plant growth (*γ* = 0.17, *P* > 0.05) (**Figure 7B**). However, the changes in the soil pH (*γ* = 0.90, *P*< 0.001) and TK (*γ* = 0.12, *P*< 0.05) under crude chitin addition indirectly promoted soybean plant growth mediated by the relative abundances of potentially beneficial microbial taxa (*γ* = 0.79, *P*< 0.001) (**Figure 7B**).

## Discussion

### Chitin amendments improved soil fertility and promoted soybean plant growth during continuous cropping

In this study, we observed that soybean growth was promoted with soil amendments of chitin (Figure S1), which was consistent with the findings reported by Debode et al. (2016). A number of studies have shown that chitin amendments imparted remarkable changes in soil physicochemical properties (Cretoiu et al., 2013; Russell, 2014; De Tender et al., 2019). The results of this study revealed that chitin amendments, especially for crude chitin, to acidic soil could significantly increase soil pH, indicating that chitin amendments are an effective method to mitigate soil acidification induced by continuous cropping of soybean (Yuan et al., 2021). The increase in soil pH and TN content could be attributed to the direct effects of chitin, as chitin, an alkaline polysaccharide, is rich in carbon and nitrogen (Xu et al., 2005; Shamshina et al., 2020). The increase in available nutrients in this study, such as the soil AP and AK contents, may be largely associated with the increased abundance of phosphate- and potassium-solubilizing microbes under chitin amendments. This is substantiated by the increase of *Streptomyces*, *Sphingomonas* and *Bacillus* in this study (**Tables S9**, **S10**), which have the ability to solubilize phosphorus and potassium (Chen et al., 2006; Xu et al., 2021). A surprising finding was that high dosages of crude chitin resulted in a substantial increase in soil ${\text{NO}}_{3}^{-}$ -N content (**Table 1**). We speculate that two reasons possibly contributed to this result. A possible interpretation of this finding could be that chitin is decomposed into plant available nitrogen (N) by increased chitin-degrading bacteria (Mendes et al., 2011; Cretoiu et al., 2013), which could further activate ammonia-oxidizing bacteria (AOB) in soils, thus resulting in the accumulation of soil ${\text{NO}}_{3}^{-}$ -N content by nitrification mediated by AOB (Kowalchuk and Stephen, 2001; De Tender et al., 2019). This finding is supported by the results in our study that the relative abundance of ammonia-oxidizing bacteria of BOTU8616 (*Nitrosospira multiformis*) was significantly increased after high dosages of crude chitin addition (**Table S10**). An additional explanation may be that the decrease in the relative abundance of denitrifying bacteria in this study, such as BOTU9715 (*Bradyrhizobium liaoningense*), could also contribute to the increase in soil ${\text{NO}}_{3}^{-}$ -N content (**Table S10**), which was concordant with the view of De Tender et al. (2019). In short, our results revealed that chitin amendments added to acidic soil improved soil chemical properties and further affected the soil microbial composition and diversity.

### Chitin amendments reduced the bacterial and fungal diversity and changed their community structure

It has been reported that the low C:N of chitin stimulates hydrolase production, which thus causes chitin added to soil to decompose rapidly and releases substantial nitrogen for microbial growth (Cretoiu et al., 2013; Koivunen et al., 2018). This could account for the increase in soil microbial activity in PC and CC when nitrogen-rich chitin was added in this study (**Figure S1E**). Debode et al. (2016) and Inderbitzin et al. (2018) found that microbial α-diversity presented no significant differences between chitin-amended and unamended soils. However, Cretoiu et al. (2014) observed that chitin amendments added to soils could promote soil microbial diversity. Conversely, Kielak et al. (2013) detected that bacterial diversities decreased after chitin addition, likely because of the particular selection of new habitat conditions created by chitin amendments to the microbial groups. In this study, we also observed that pure and crude chitin amendments decreased bacterial and fungal α-diversity in both bulk and rhizosphere soils (**Figure S2**). Therefore, we speculate that the lower diversity in PC and CC was mainly due to the larger copiotrophic bacteria (r-strategists) exhibiting higher growth rates than oligotrophic species (k-strategists) when the N resources increased under chitin amendments.

We found that the community structures of pure and crude chitin-amended treatments in both bulk and rhizosphere soils were clearly different from those of the unamended soils (**Figure 1B**). It was generally reported that the bacterial and fungal communities in bulk soils were well differentiated from those in rhizosphere soils, which agrees with the concept that the enrichment of root exudates can influence rhizosphere microbial communities (Chaparro et al., 2014; Liu et al., 2018). However, this phenomenon was not detected in this study. We found that the impacts of chitin amendments on soil bacterial and fungal community structures were stronger than those of the rhizosphere effect, as the bacterial and fungal communities in pure and crude chitin-amended soils were obviously divided into two separate groups (**Figure 1A**, **Table S3**). In contrast, Deng et al. (2021) and Liu et al. (2018) observed that chemical and organic fertilizers or bio-organic fertilizer addition exerted weaker effects on microbial community structures than the rhizosphere effect. The stronger disturbances of chitin on microbial community structures compared to other organic fertilizers could be attributed to nitrogen stress and movement in soils of chitin as nitrogen-rich and slow-release fertilizer (Xu et al., 2005; Deng et al., 2021).

### Chitin amendments increased the abundances of potentially beneficial microbes but decreased the abundances of potential plant pathogens

Mounting studies have documented that chitin amendments can activate specific microbial populations that are conducive to plant growth and antagonistic to potential plant pathogens (Bharathi et al., 2004; Rajkumar et al., 2008; De Tender et al., 2019). In this study, the relative abundances of BOTU6992 (*S. limnosediminicola*), BOTU8821 (*S. lutea*), and BOTU8988 (*S. palustris*), which are affiliated with the *Sphingomonas* genus, were markedly higher in pure and crude chitin treatments than in C0B in both bulk and rhizosphere soils (**Figure 3**, **Tables S9**, **S10**). BOTU10033 (*S. aureus*), BOTU10262 (*S. avermitilis*), BOTU4104 (*S*. cyaneus), BOTU7747 (*S*. *asenjonii*) and BOTU8104 (*S. rutgersensis*) were identified as *Streptomyces* (**Table S10**), and BOTU10180 (*Bacillus vireti*), BOTU10233 (*Bacillus aryabhattai*), and BOTU10326 (*Bacillus simplex*) were affiliated with *Bacillus*, which were significantly increased in the crude chitin-treated soils (**Table S10**). *Sphingomonas*, *Streptomyces* and *Bacillus* were reported to have the capability to solubilize phosphorus and potassium (Chen et al., 2006; Xu et al., 2021) and inhibit soil-borne pathogens (Durán et al., 2018; Cobo-Díaz et al., 2019). Thus, the aforementioned increase in those bacterial taxa might be associated with antagonistic activity against potential plant pathogens and the improvement of soil nutrients. In addition, BOTU3878 and BOTU9424 were classified as *Mesorhizobium huakuii* and *Nitrospira moscoviensis*, which participate in nitrogen fixation and nitrification, respectively (Hu et al., 2021; Liu et al., 2022). The relative abundances of these species were found to be significantly higher in the pure chitin treatment (**Table S9**), implying that chitin addition could enhance soil nitrogen nutrition.

The relative abundances of FOTU674 (*Purpureocillium lilacinum*), FOTU1202 (*Penicillium infrabuccalum*), FOTU1909 (*Penicillium raperi*) and FOTU676 (*Penicillium janthinellum*) were significantly increased in PC and CC (**Figures 4B, D**, **Tables S9**, **S10**). Additionally, the relative abundances of FOTU2062 (*Metarhizium frigidum*) and FOTU843 (*Pochonia chlamydosporia*) were notably enriched in PC both in bulk and rhizosphere soils (**Table S9**). *Purpureocillium* and *Penicillium* are well-known beneficial fungi that control phytopathogenic fungi such as *Fusarium oxysporum*, which can cause soybean wilt disease and root rot disease (Wang et al., 2016; Herrera et al., 2022). *Metarhizium* can exert negative effects on soybean aphids (*Aphis glycines*), and *Pochonia chlamydosporia* is widely recognized as a promising biocontrol agent to suppress cyst and root knot nematodes (Hamid et al., 2017; Clifton et al., 2018). Therefore, we speculate that the enrichment of potentially beneficial fungi in chitin amendments may largely lead to mitigation of the pathogenic risk of potential plant pathogens. Notably, we found that chitin amendments caused a larger decrease in the relative abundance of well-known potential plant pathogens, such as FOTU2225 (*Fusarium equiseti*) and FOTU26 (*Fusarium solani*), in the bulk and rhizosphere soils (**Figures 4A, C**, **Tables S9**, **S10**). In addition, FOTU118 (*Cylindrocarpon faginatum*), FOTU501 (*Paraphoma radicina*) and FOTU616 (*Septoria epilobii*) were identified as key ‘pathogen facilitators’ that aid in pathogen invasion (Tao et al., 2021) and cause root rot and brown spot diseases of legumes (Cruz et al., 2010; Dang et al., 2022). Thus, the increase in beneficial fungi and the decrease in potential plant pathogens provided evidence that chitin addition could contribute to the healthy development of the soil microbiota.

### Chitin amendments induced a simpler pathogen-associated subnetwork

The subnetworks were reconstructed from the global networks, which reflected the interactions between potential plant pathogens and their connected microbial taxa (**Figures 5**, **6**). We found that chitin amendments formed simpler pathogen-associated subnetwork structures with lower average node degrees and less cooperation between pathogens and their connected microbial taxa, which indicated that chitin amendments could help mitigate the destructive effects of potential plant pathogens (Sato et al., 2010; Kielak et al., 2013). The interaction between *Fusarium* and *Mortierella* of crude chitin-added subnetworks was negative in bulk soils but positive in rhizosphere soils (**Table S12**). This negative relationship in bulk soil was most likely because *Mortierella*, as an opportunist, could respond rapidly to emerging ecological opportunities in the soils (increased carbon sources), which could compete with *Fusarium* for nutrients and living space (Debode et al., 2016). However, this positive interaction between the bacterium *Mortierella* and the plant pathogen *Fusarium* in rhizosphere soils could be ascribed to the cooperation between *Mortierella* and pathogenic fungi in the decomposition of allelochemicals or the tight interactions facilitating fungal pathogenicity in rhizosphere soils (Wang et al., 2020a; Hu et al., 2021). Interestingly, both in bulk and rhizosphere soils, negative interactions between *Sphingomonas* and *Fusarium* emerged in the subnetworks of CC. These antagonistic interactions may be explained by the concept that Sphingomonas utilized allelochemicals (e.g., salicylic acid) as the only carbon source (Story et al., 2004), which could degrade toxic substances in soils and thus suppress potential pathogens (Macchi et al., 2018). Thus, the simpler pathogen-associated subnetwork structures and antagonistic interactions against pathogens suggested that chitin amendments could mitigate the potential transmission of potential plant pathogens and inhibit their ecological function.

### Abiotic and biotic factors jointly promoted soybean plant growth under chitin amendments

Changes in soil physicochemical properties could directly affect potential plant pathogens to enhance the soil disease-suppressive ability (Bongiorno et al., 2019). However, they mainly indirectly affected potential plant pathogens by influencing soil microbial assemblies and in turn improved soil disease suppression (Shen et al., 2019; Hu et al., 2021). In this study, we observed that the increase in soil pH under chitin amendments mainly indirectly affected beneficial microbes and soil microbial activities and further suppressed potential plant pathogens to promote soybean growth (**Figure 7**). The microbiological mechanisms of disease-suppressive soils were reported to be different and mainly included ‘general suppression’ and ‘specific suppression’ (Weller et al., 2002). ‘General suppression’ is commonly associated with higher soil microbial activities, and ‘specific suppression’ is mainly attributed to the dominance of specific microbial populations (Janvier et al., 2007). In our study, SEM showed that pure chitin amendments markedly increased soil microbial activity by increasing the soil pH, which thus promoted soybean plant growth (**Figure 7A**). This is mainly because pure chitin addition increased the numbers and activities of chitin-degrading organisms, which could suppress potential plant pathogens by decomposing chitin-rich cell walls (Mendes et al., 2011; De Tender et al., 2019). This strategy belonged to ‘general suppression’, whose disease-suppressive ability was untransferable and dependent on soil organic matter content (Janvier et al., 2007). Interestingly, we found that the increase in soil pH mediated by crude chitin addition indirectly promoted soybean growth by increasing the populations of potentially beneficial microbes (**Figure 7B**). This may because crude chitin addition recruited multiple specific beneficial microbial populations (**Figure 7B**), which could suppress potential plant pathogens by competing for nutrients or colonization space (Rajkumar et al., 2008; Debode et al., 2016). This strategy belonged to ‘specific suppression’ with more stable and long-lasting disease-suppressive ability (Weller et al., 2002). These findings confirmed that chitin, particularly crude chitin, as a promising soil amendment could effectively alleviate soybean continuous cropping obstacles by developing disease-suppressive soils.

## Conclusions

We investigated the impacts of chitin amendments on soil proprieties, microbial communities and soybean growth in a soil with continuous cropping soybean for 5 years. We found that both PC and CC greatly increased soil pH and available nutrient contents and improved soybean plant growth and soil microbial activities (FDA). Chitin amendments remarkably enriched potentially beneficial bacterial and fungal taxa but reduced potential plant pathogens, implying that chitin addition may contribute to the development of healthy soil microbiota. In addition, chitin application caused looser pathogenic subnetwork structures, which demonstrated that chitin amendments may mitigate the spread and ecological risks of potential plant pathogens in soil–plant systems. Interestingly, PC promoted soybean growth by increasing the numbers and activities of chitin-degrading organisms to decompose pathogenic cell walls, which belonged to general disease suppression. CC improved soybean growth by recruiting specific beneficial microbial taxa to compete for nutrients and colonization space with potential plant pathogens, which was identified as specific disease suppression. In conclusion, chitin amendments, especially CC, might be an essential approach to mitigate continuous soybean cropping obstacles by suppressing soil-borne diseases.

## Data availability statement

The datasets presented in this study can be found in online repositories. The names of the repository/repositories and accession number(s) can be found in the article/**Supplementary Material**.

## Author contributions

YF: Investigation, visualization, formal analysis, writing – original draft. JL: Supervision, fund acquisition, experiment design, writing – reviewing & editing. ZL: Visualization, formal analysis. XH: Formal analysis, data curation. ZY: Formal analysis, investigation. YL: Formal analysis, investigation. XC: Formal analysis, investigation. LL: Conceptualization, methodology. JJ: Conceptualization, methodology. GW: Conceptualization, methodology, writing – review & editing. All authors contributed to the article and approved the submitted version.

## Funding

This study was financially supported from National Natural Science Foundation of China (42177105), the Strategic Priority Research Program of the Chinese Academy of Sciences (XDB28070302), the Key Research Program of Frontier Sciences, Chinese Academy of Sciences (ZDBS-LY-DQC017), Heilongjiang Province Scientific Research Fund of Provincial Scientific Research Institutes (CZKYF2022-1-B014) and Heilongjiang Provincial Natural Science Foundation of China (ZD2022D001).

## Conflict of interest

The authors declare that the research was conducted in the absence of any commercial or financial relationships that could be construed as a potential conflict of interest.

## Publisher’s note

All claims expressed in this article are solely those of the authors and do not necessarily represent those of their affiliated organizations, or those of the publisher, the editors and the reviewers. Any product that may be evaluated in this article, or claim that may be made by its manufacturer, is not guaranteed or endorsed by the publisher.
