# Supplementary material for: Chitin amendments eliminate the negative impacts of continuous cropping obstacles on soil properties and microbial assemblage
Source: Front Plant Sci. 2022 Nov 24;13:1067618. doi: 10.3389/fpls.2022.1067618 (PMC9730418; doi:10.3389/fpls.2022.1067618)
Supplement: Supplementary file 1 [file DataSheet_1.docx]

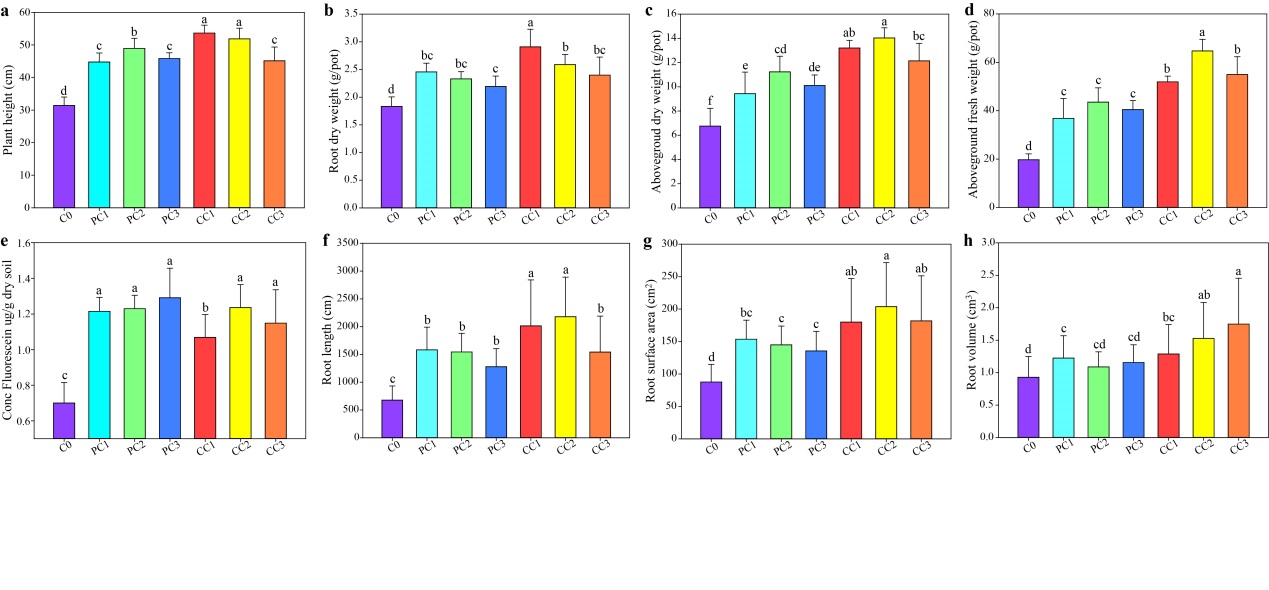


**Fig S1** Effects of pure and crude chitin amendments on soybean plant height (a), root dry weight (b), aboveground dry weight (c), aboveground fresh weight (d), bulk soil microbial activities (e), root length (f), root surface area (g) and root volume (h). Different letters indicate significant differences among treatments (*P* < 0.05). The treatment description is same as Fig 1.


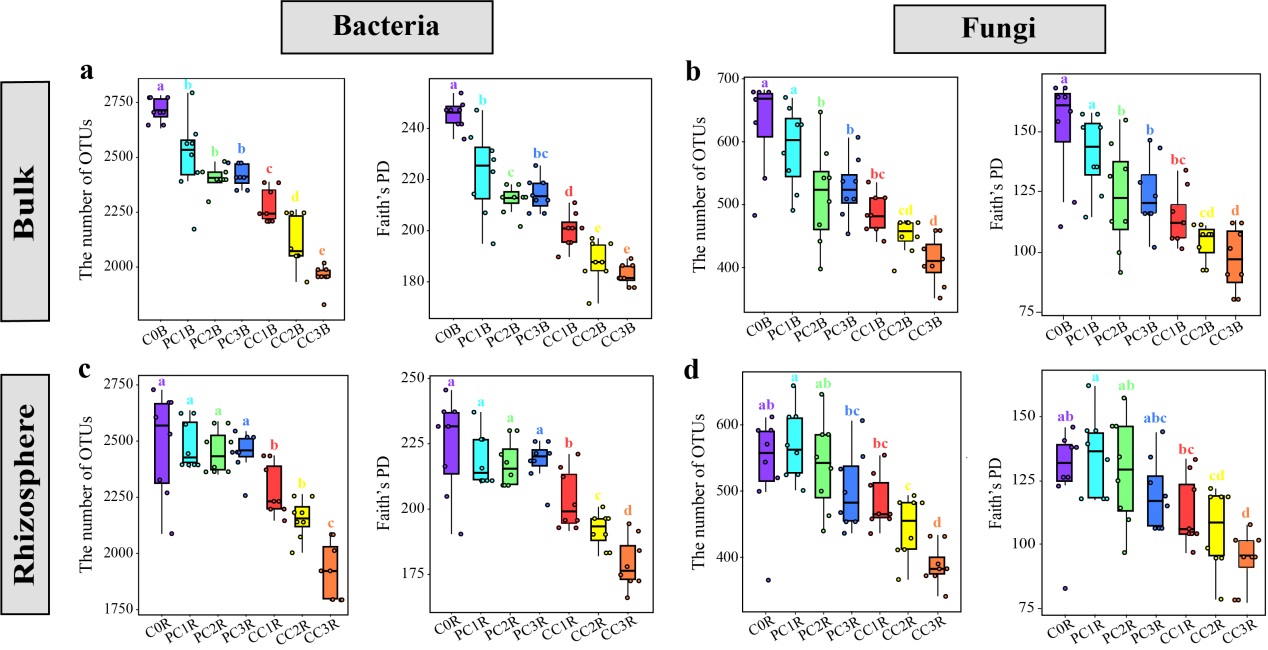


**Fig S2** The bacterial (a and c) and fungal (b and d) diversity indexes of the total number of OTUs and the Faith's phylogenetic diversity (PD) in the bulk and rhizosphere soils. Different letters indicate significant differences among treatments (*P* < 0.05). The treatment description is same as Fig 1.


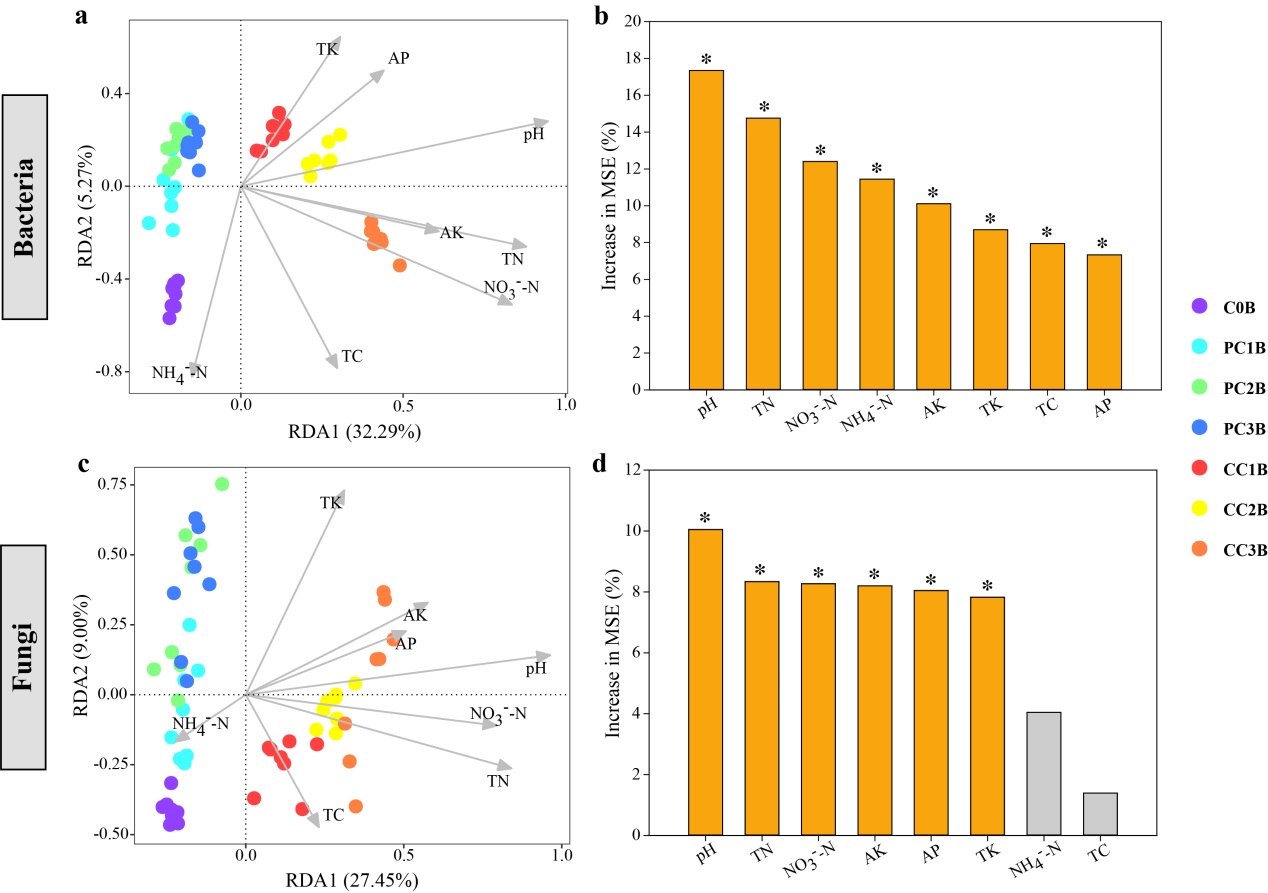


**Fig S3** Redundancy analysis (RDA) and random forest analysis showing the correlation between soil properties and bacterial (a and b) and fungal (c and d) community structures in the bulk soils. TC, TN and TK represent soil total carbon, total nitrogen and total potassium, respectively; AP and AK represent soil available phosphorus and available potassium, respectively. Significance levels are: **P* < 0.05. The treatment description is same as Fig 1.


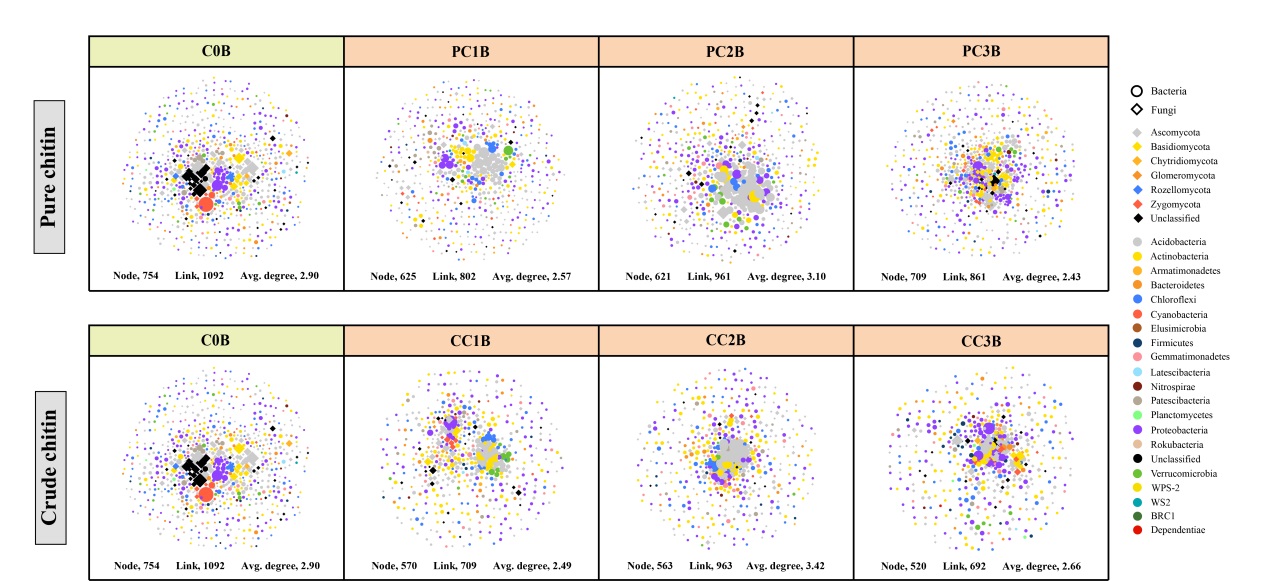


**Fig S4** Microbial network graphs under different pure and crude chitin addition dosages in the bulk soils. Circles and diamonds represent bacteria and fungi, respectively. Each node represent an OTU; the size of each node is proportional to degree; the nodes colored by taxonomy. Avg. degree represents average node degree, which is the number of edges of a node to others. Edges represent significant interactions. Blue and red lines indicated positive and negative interactions between two individual nodes, respectively. The treatment description is same as Fig 1.


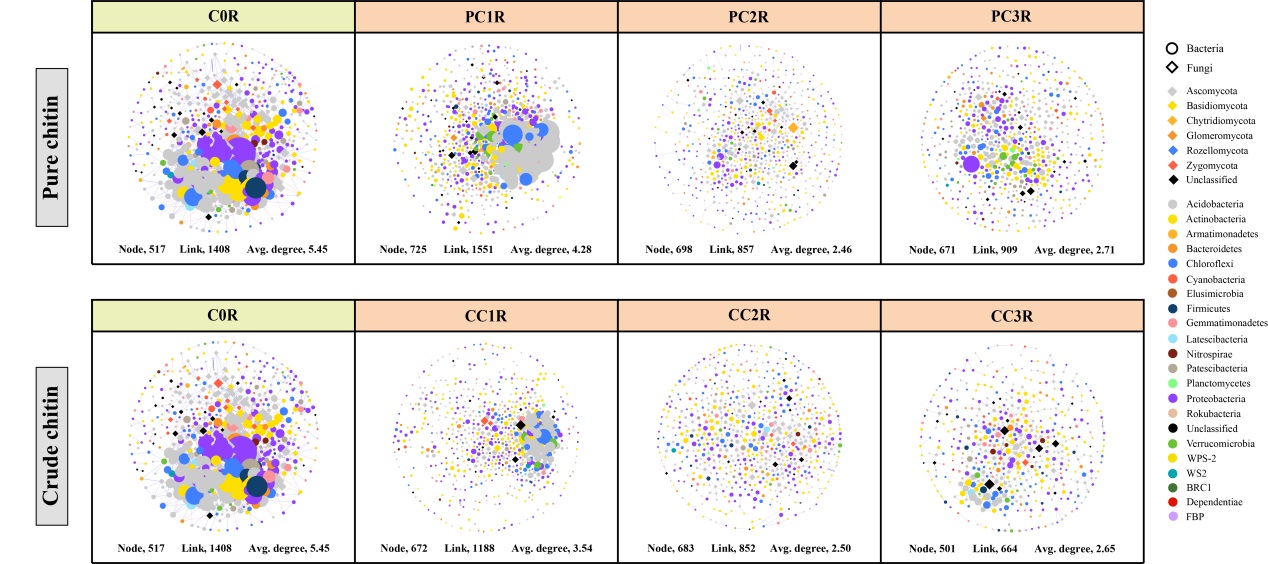


**Fig S5** Microbial network graphs under different pure and crude chitin addition dosages in the rhizosphere soils. Circles and diamonds represent bacteria and fungi, respectively. Each node represent an OTU; the size of each node is proportional to degree; the nodes colored by taxonomy. Avg. degree represents average node degree, which is the number of edges of a node to others. Edges represent significant interactions. Blue and red lines indicated positive and negative interactions between two individual nodes, respectively. The treatment description is same as Fig 1.
